# Effectiveness of non-bedside teaching during the COVID-19 pandemic: a quasi-experimental study

**DOI:** 10.1186/s12909-022-03141-z

**Published:** 2022-01-31

**Authors:** Henrik Heitmann, Philipp Wagner, Elisabeth Fischer, Martin Gartmeier, Friederike Schmidt-Graf

**Affiliations:** 1 Department of Neurology, School of Medicine, Technical University of Munich (TUM), Munich, Germany; 2 TUM Medical Education Center, School of Medicine, TUM, Munich, Germany

**Keywords:** COVID-19, Teaching, Clinical Competence, Neurology

## Abstract

**Background:**

The COVID-19 pandemic poses a huge challenge for clinical teaching due to contact restrictions and social distancing. Medical teachers have to balance potential risks and benefits of bedside teaching, especially in course formats intended to foster practical clinical skills. In this context, we aimed to address the question, whether presence-based teaching formats without patient involvement are suitable to teach practical skills.

**Methods:**

In this quasi-experimental study, presence-based teaching formats with and without patient contact were retrospectively compared regarding their effects on medical students’ theoretical knowledge and practical skills, i.e. the performance and clinical interpretation of the neurological exam. To this end, evaluations from 102 students and their lecturers participating in a neurological bedside teaching course at a German university hospital between October 2020 and April 2021 were obtained. Students were initially randomly assigned to course dates. However, 53 students assigned to courses in November and December 2020, were not able to go bedside due to contact restrictions. These students formed the interventional group and the remaining 49 students the control group. The primary outcome measures were students’ overall grading of the course (school grades, 1–6) as well as ratings of knowledge and skills provided by the students themselves and their lecturers on a numerical rating scale (0–10). Comparison between groups was performed using frequentist and Bayesian t-statistics.

**Results:**

The teaching format without patient contact received a significantly poorer overall grade by the students (*p* = 0.018). However, improvements in the students’ self-ratings of knowledge and skills did not differ between the two formats (all *p* > 0.05, BF_10max_ = 0.42). Moreover, especially practical skills were even rated significantly better in the group without patient contact by the lecturers (*p* < 0.001).

**Conclusions:**

Teaching formats without patient contact are less well-received by the students. However, they are able to teach practical skills regarding the performance and clinical interpretation of examination techniques. Still, the evaluations obtained might not adequately capture the importance of bedside teaching in preparing future physicians for their practice. Perspectively, hybrid teaching approaches including flipped-classroom concepts hold considerable potential to enhance effectiveness of bedside teaching in the present pandemic situation and in the future.

## Background

The ongoing COVID-19 pandemic has strongly impacted the education of undergraduate medical students [1]. Due to contact restrictions, entire curricula had to be transferred to distance learning at short notice [2]. Through e-learning courses, lecturers can quite easily replace information-focused teaching formats with few interactive elements like lectures. However, for courses in which medical students acquire practical skills, creating alternative course formats is much more difficult [3, 4]. Due to this difficulty, many clinical placements, especially bedside teaching courses, were suspended in medical schools [5], whereby many Neurological courses were affected too [6]. In addition to these organizational issues the present pandemic situation is associated with substantial emotional burden for students [7] and clinical teachers working as healthcare providers [8–10].

In Neurology and other clinical disciplines bedside teaching has fundamental importance in fostering core clinical skills, such as hypothesis driven physical examinations and clinical reasoning [5, 11]. Hence, in view of the ongoing pandemic, medical teachers find themselves challenged to balance the benefits of bedside teaching for their students and the corresponding potential risks for their patients [12]. Thus, the question whether presence-based teaching formats with no patient involvement are suitable to teach practical skills is an urging one for clinical teachers in Neurology and beyond.

To this end, we compared presence-based teaching with and without patient contact regarding its effects on medical students’ theoretical knowledge and practical skills, i.e. the performance and clinical interpretation of the neurological exam.

## Methods

### Participants

All fourth-year medical students scheduled to participate in a presence-based neurological bedside teaching course, as part of their curriculum, between October 2020 and April 2021 (*n* = 104) were eligible for inclusion into the study. There were no further exclusion criteria. The initial assignment of the students to course dates was randomized. However, due to contact restrictions, students assigned to course dates in November and December 2020 (*n* = 54) were not able to go bedside during the course due to contact restrictions. These students, thus formed the interventional group for the present study. The remaining students, that were assigned to course dates in October 2020 and April 2021 (*n* = 50) were able to participate in a regular course including patient contact and thus served as the control group. All students were asked to voluntarily evaluate the course and subsequently 102/104 (response rate 98%) of them anonymously completed evaluation forms at the beginning and end of the course. Of those, 49 were included the control group and 53 in the interventional group. The present study therefore had a quasi-experimental design and retrospectively analyzed data from an interventional study that evaluates different teaching formats during the COVID-19 pandemic and was approved by the ethics committee of the Medical Faculty of the Technical University of Munich (TUM). The study was performed in accordance with the relevant guidelines and regulations.

### Bedside teaching

The neurological bedside teaching at TUM School of Medicine takes place in the fourth year, which is the students’ second year of clinical training. In advance, students received a script providing background information on the performance and clinical interpretation of the neurological exam to prepare for the course. Subsequently, students attended an in-person teaching session in small groups of maximum three students per lecturer for three hours on one afternoon. All lecturers were resident physicians at the TUM Department of Neurology. At the beginning of the course, lecturers gave a short introduction on the theoretical backgrounds of the neurological exam. This included repeating the basics of functional neuroanatomy and their implications for basic principles of the neurological exam, such as the importance of symmetry. Moreover, students were provided with a structured approach on how to perform, interpret and report the neurological exam. Additionally, the importance of history taking in guiding the clinical examination procedure and other clinical aspects were emphasized. After that, the lecturers demonstrated how to perform a neurological exam and students had time to practice the neurological exam on their peers under close supervision. Subsequently, students usually go to the bedside to perform history taking and a full neurological exam on patients. Afterwards, students present the patient´s cases to the lecturers and pathological findings in the neurological exam are re-assessed together. As previously mentioned, only the students in the control group were able to go bedside but not the interventional group, who thus had additional time to practice the neurological exam on their peers.

### Evaluations

Evaluations were obtained at two different time points, before (T0) and after the course (T1).

Students were asked to rate different aspects of their respective theoretical knowledge and their practical skills regarding the performance and interpretation of the neurological exam, using identical evaluation forms at T0 and T1. Ratings were obtained using numerical rating scales (NRS) from 0 (“not good at all”) to 10 (“very good”). For the theoretical knowledge, items covered were *functional neuroanatomy* (e.g. knowing the names of the cranial nerves and their physiological function; being able to differentiate upper and lower motor neuron signs), *systematology* (e.g. knowing into which parts the neurological exam can be divided and the best sequence to assess them), *basic principles* (e.g. knowing the importance of symmetry when performing a neurological exam and how to differentiate physiological from pathological findings) as well as *quantification* (e.g. knowing how to grade muscle strength or reflexes). Items regarding practical skills were *examination skills* (e.g. ability to autonomously perform a neurological screening exam), *information transfer* (e.g. ability to use information derived from history taking to focus the examination procedure), *documentation* (e.g. ability to document findings in a structured manner), *oral presentation* (e.g. ability to communicate findings in a structured manner), and *red flags* (e.g. ability to identify alarm signs during the examination).

Moreover, students were asked to provide an overall grade for the course from 1 (“very good”) to 6 (“insufficient”) at T1. Additionally, students could provide free-text comments including suggestions for improvement at T1. Furthermore, lecturers were asked to rate the average level of their students’ group previous theoretical knowledge as well as the practical skills acquired from 0 (“not good at all”) to 10 (“very good”), again using NRS, at T1.

## Statistics

Statistical analyses were performed using JASP (JASP Team 2021, Version 0.15). To compare the students’ self-evaluations of knowledge and skills at baseline (T0) and follow-up (T1) paired sample t-tests were applied. To compare changes in these evaluations as well as overall ratings by students and lecturers between the group with and without patient contact t-tests for independent samples, including estimates of effect size (Cohen´s d) and the respective 95% confidence intervals, were used. Change in evaluations was calculated by subtracting T0 from T1 values. Values of d = 0.2, 0.5 and 0.8 were considered indicative of small, medium and large effect sizes, respectively. Additionally, Bayesian t-tests were performed when results did not differ between the groups, again using JASP, to further evaluate the potential significance of these negative findings. In Bayesian hypothesis testing the Bayes factor (BF_10_) provides a continuous measure to quantify the evidence in favor of or against a certain hypothesis. It thereby especially allows to interpret the significance of negative findings (“absence of evidence vs. evidence of absence”) [13]. A BF_10_ > 1 indicates more evidence for the alternative hypothesis, whereas a BF_10_ < 1 provides evidence against the alternative hypothesis. A BF_10_ < 0.33 and < 0.1 is regarded as moderate and strong evidence against the alternative hypothesis, respectively. A sensitivity analysis for independent sample t-statistics using G*Power [14] showed that using an error probability (α) of 0.05 and a power (1-ß error probability) of 0.8 the present sample size was sufficient to detect potential group differences with medium effect sizes (Cohen´s d = 0.56). The significance level for all statistical tests was set to 0.05 two-tailed.

## Results

### Overall grading of the course by the students

A total of 102 students were asked to provide an overall grade for the course, 49 in the control and 53 in the interventional group. Students in the control group, with patient contact, graded the course significantly better than those in the interventional group without patient contact (mean ± standard deviation (SD) 1.19 ± 0.6 vs. 1.54 ± 0.96, t_df (98)_ = 2.4, p = 0.018), with a medium effect size (Cohen´s d = -0.47, 95% confidence interval (CI) = [-0.87,-0.07]).

### Self-ratings of knowledge and skills by the students

Students rated their respective theoretical knowledge and their practical skills regarding different aspects of the neurological exam at the beginning (T0) and end (T1) of the course. There was a highly significant increase in knowledge and skills from T0 to T1 in both groups for all items (all p < 0.001). However, neither changes in knowledge nor changes in skills differed significantly between the groups with and without patient contact (all p > 0.05). This was confirmed by Bayesian hypothesis testing, that provided moderate to strong evidence against a difference between the two groups (for detailed test statistics please see Table 1).

**Table 1:** Students’ self-ratings of knowledge and skills

	**Items**	**With patient contact** *(mean* ± *SD)*		*Paired sample t-tests*	**Without patient contact** *(mean* ± *SD)*		*Paired sample t-tests*	*Independent sample t-tests to compare changes (*Δ) *between the two groups*
		*T0*	*T1*	*T0 vs. T1*	*T0*	*T1*	*T0 vs. T1*	*Δ CG vs. Δ IG*
**Know-ledge**	*Functional neuroanatomy*	6.35 ± 1.5	8.20 ± 1.4	t_df (48)_ = -8.1 p < 0.001	6.60 ± 1.7	8.38 ± 1.1	t_df (51)_ = -7.6 p < 0.001	t_df (99)_ = 0.21, p = 0.836, BF_10_ = 0.21 d = -0.04, 95% CI = [-0.35, 0.43]
	*Systematology*	4.84 ± 2.1	8.57 ± 1.3	t_df (48)_ = -11.0 p < 0.001	4.50 ± 2.5	8.85 ± 1.2	t_df (51)_ = -12.3 p < 0.001	t_df (99)_ = -1.24, p = 0.217, BF_10_ = 0.42 d = -0.25, 95% CI = [-0.64, 0.15]
	*Basic principles*	4.69 ± 1.8	8.49 ± 1.2	t_df (48)_ = -12.7 p < 0.001	4.65 ± 2.0	8.60 ± 1.3	t_df (51)_ = -13.0 p < 0.001	t_df (99)_ = -0.34, p = 0.732, BF_10_ = 0.22 d = -0.07, 95% CI = [-0.46, 0.32]
	*Quantification*	3.35 ± 2.0	7.65 ± 1.7	t_df (48)_ = -12.5 p < 0.001	3.31 ± 2.4	8.00 ± 1.4	t_df (51)_ = -13.1 p < 0.001	t_df (99)_ = -0.77, p = 0.441, BF_10_ = 0.27 d = -0.15, 95% CI = [-0.55,0.24]
**Skills**	*Examination*	2.94 ± 2.2	7.65 ± 1.5	t_df (47)_ = 14.8 p < 0.001	3.28 ± 2.5	7.87 ± 1.5	t_df (52)_ = 12.6 p < 0.001	t_df (99)_ = 0.25, p = 0.801, BF_10_ = 0.22 d = 0.05, 95% CI = [-0.34, 0.44]
	*Information transfer*	4.54 ± 2.3	7.83 ± 1.4	t_df (47)_ = -10.5 p < 0.001	4.75 ± 2.2	7.55 ± 1.4	t_df (52)_ = -9.0 p < 0.001	t_df (99)_ = 1.1, p = 0.261 BF_10_ = 0.37 d = 0.23, 95% CI = [-0.17, 0.62]
	*Documentation*	3.81 ± 2.5	6.69 ± 1.5	t_df (47)_ = -8.8 p < 0.001	4.02 ± 2.2	6.57 ± 1.6	t_df (52)_ = -9.8 p < 0.001	t_df (99)_ = 0.79, p = 0.432, BF_10_ = 0.28 d = 0.16, 95% CI = [-0.23, 0.55]
	*Oral presentation*	3.85 ± 2.1	6.88 ± 1.5	t_df (47)_ = -9.4 p < 0.001	4.47 ± 2.1	7.11 ± 1.6	t_df (52)_ = -9.2 p < 0.001	t_df (99)_ = 0.88, p = 0.380, BF_10_ = 0.30 d = 0.18, 95% CI = [-0.22, 0.57]
	*Red flags*	3.96 ± 2.3	7.40 ± 1.6	t_df (47)_ = -10.8 p < 0.001	4.23 ± 2.0	7.40 ± 1.6	t_df (52)_ = -11.0 p < 0.001	t_df (99)_ = 0.63, p = 0.533, BF_10_ = 0.25 d = 0.13, 95% CI = [-0.27, 0.52]

The table shows the results from paired sample t-tests to assess changes from T0 to T1 within groups as well as frequentist and Bayesian t-tests for independent samples comparing the change in the different parameters between the control group (with patient contact) and the interventional group (without patient contact)

*SD* standard deviation, *T0* baseline evaluation before the course, *T1* follow-up evaluation after the course, Δ T1-T0, *d* Cohen´s d, *CI* confidence interval, *BF*_*10*_ Bayes factor

### Ratings of knowledge and skills by the lecturers

**Fig. 1:**
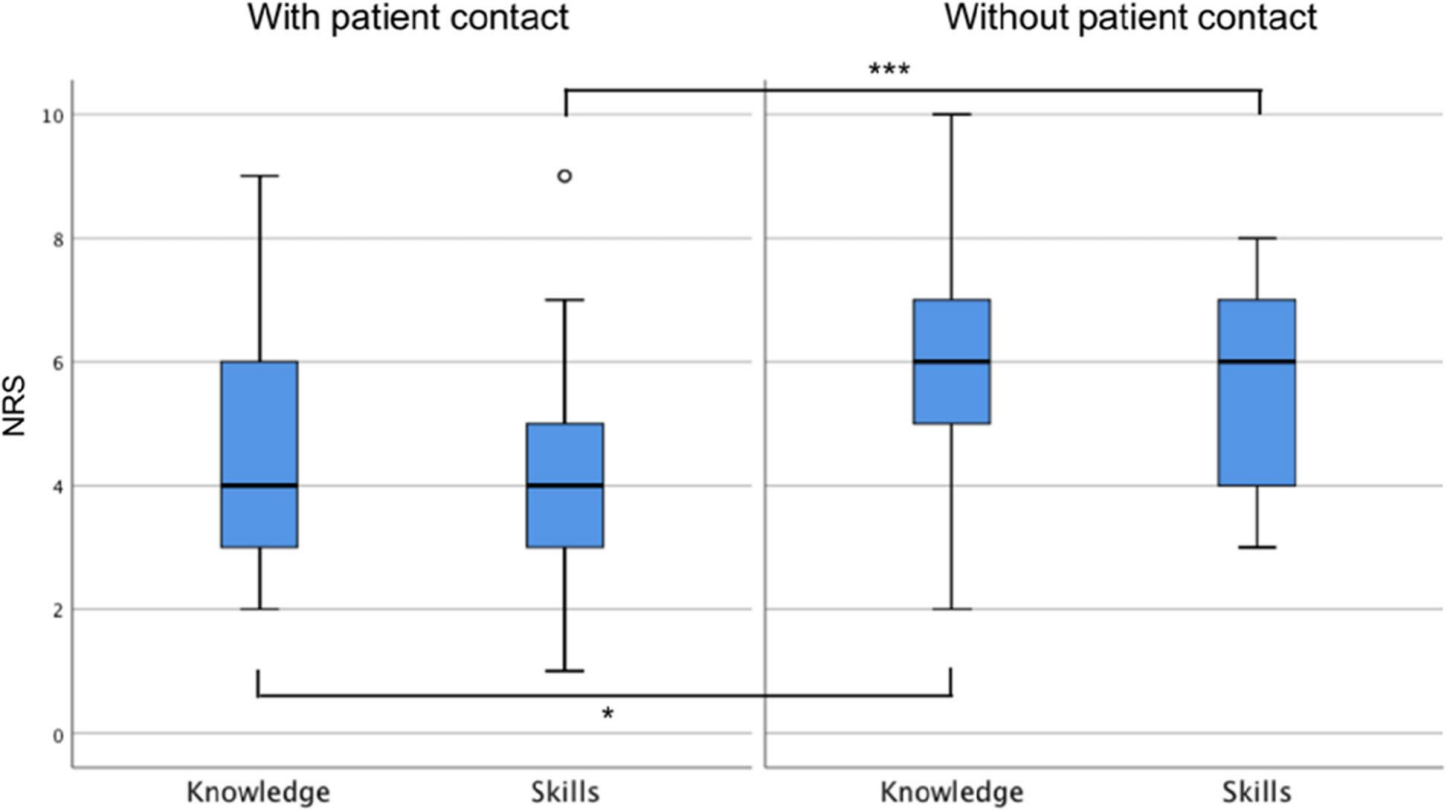
Comparison of lecturer ratings for theoretical knowledge and practical skills. The figure graphically illustrates the comparison of lecturer ratings for previous theoretical knowledge and practical skills acquired during the course between the control group (with patient contact) and the interventional group (without patient contact). Bars depict the median. Lower and upper limit of boxes depict the 25^th^ and 75^th^ percentile respectively. Whiskers depict the range of results and dots represent outliers. NRS = Numerical rating scale. *p<0.05, ***p<0.001

Lecturers were asked to provide average ratings of previous theoretical knowledge and the skills acquired during the course for their respective small group of students. Ratings of knowledge were significantly higher in the interventional group without patient contact than in the control group with patient contact (mean ± SD 4.9 ± 2.2 vs. 6.0 ± 2.0, t_df (100)_ = -2.6, p = 0.011), with a medium effect size (Cohen´s d = -0.51, 95% CI = [-0.91,-0.12]). Moreover, lecturers rated the practical skills acquired even higher in the interventional group without patient contact compared to the control group with patient contact (mean ± SD 4.35 ± 1.9 vs. 5.7 ± 1.7, t_df (100)_ = -3.9, p < 0.001), with a large effect size (Cohen´s d = -0.77, 95% CI = [-1.17,-0.36]) (for a graphical illustration of results please see Fig. 1).

### Free-text comments from students

Students from both groups commented that they very much appreciated the possibility to attend an in-person “hands-on” teaching format despite the pandemic situation.

In the group with patient contact, students further explicitly stated, that they valued the possibility to go bedside to practice and perform the neurological exam in a clinical context. Moreover, these students recommended to provide additional videos of examination techniques that could be used to prepare themselves for the course and to recapitulate afterwards.

In the group without patient contact, students explicitly stated that they understand the safety measure of not going bedside to protect the patients. However, they also commented, that they felt like not going bedside hampers the clinical application of the skills acquired.

## Discussion

In the present study, ratings of theoretical knowledge and practical skills from students participating in two clinical teaching formats, with and without patient contact, and their lecturers were assessed and compared. The results show, that the teaching format without patient contact received a poorer overall grade by the students despite being equally effective in improving their theoretical knowledge and practical skills as assessed by self-ratings. Additionally, the students’ theoretical knowledge and practical skills were rated significantly better in the group without patient contact by the lecturers. However, students commented that not going bedside during the course hampers their ability to apply the practical skills acquired in a clinical context.

The poorer overall grade for the format without patient contact likely reflects that students perceive going bedside as an integral part of medical education [4, 11]. This notion is supported by the students’ free-text comments in the present study and a recent literature review on virtual replacement formats for clinical placements during the COVID-19 pandemic [4]. This highlights the critical role of in-person bedside teaching formats for the students’ motivation and their ability to transfer the knowledge and skills acquired [4]. Clinical teachers, also in the field of Neurology, have described many creative efforts, to compensate for missing clinical placements during the COVID-19 pandemic e.g. by using online real-time patient contact for history taking and guiding clinical examinations in virtual formats [15, 16]. Such possibilities to interact with patients and to apply clinical knowledge to real cases also in e-learning formats is very much appreciated by students [17]. Moreover, encouraging experiences regarding online teaching of other important aspects of bedside teaching including clinical reasoning have been reported [18, 19]. Still, teaching practical skills in a clinical context poses a huge challenge to medical teachers in the present pandemic situation [4]. In the present study students explicitly commented how grateful they were for being able to participate in an in-person “hands on” teaching format, even without patient contact. Thus, in view of the ongoing COVID-19 pandemic medical teachers find themselves in the difficult situation to balance the benefits for their students and risks for their patients, when performing in-person bedside teaching [12]. In this context, the present results provide evidence that certain practical clinical skills, such as examination techniques, can be effectively taught also without patient contact. Unfortunately, the literature comparing teaching formats for physical examination techniques with and without patient contact thus far is sparse, very heterogeneous in methodology and yielded partially conflicting results [20, 21]. The present findings did not show a significant difference of self-ratings for theoretical knowledge and practical skills when comparing student groups with and without patient contact. The lack of such a difference regarding improvements in the students’ theoretical knowledge in the present study might be attributed to the fact that both groups received comparable input on theoretical aspects by their lecturers. The more surprising finding is likely the lack of a difference regarding the improvement in practical skills, that were even rated higher in the group without patient contact by the lecturers. This finding might be explained by the strong clinical emphasis of the course, also in the format without patient contact, which was shown to be of particular importance to successfully teach the neurological exam [22]. Moreover, in both groups students were able to examine and observe their peers in a small group setting, which has also been identified as an important factor to successfully acquire clinical examination techniques [23]. However, several limitations have to be taken into account when interpreting the present results. First, the items used for the evaluations likely do not adequately cover important “soft skills” such as communication, empathy and humanism that are crucial for doctor-patient relationships and are primarily developed through patient contact [5]. Second, the present findings are largely based on subjective ratings and not on standardized performance assessments. However, the very nature of these subjective ratings provides important insights on the students’ perception, especially in these pandemic times. Third, the better lecturer ratings of practical skills in the interventional group might be partially attributable to the additional time these students had to practice the neurological exam on their peers. However, this was not reflected by the corresponding self-ratings. Fourth, the better lecturer ratings of previous theoretical knowledge in the group without patient contact might reflect a better individual preparation by the students in this group. Higher levels of previous theoretical knowledge were found to enable a more effective transfer of practical skills during in-person bedside teaching [24]. Hence, more efforts should be put into fostering students’ theoretical knowledge in preparation for in-person teaching formats and future studies should further evaluate this relationship.

## Conclusions

In conclusion, our results show that non-bedside teaching appears to be an effective approach for teaching practical clinical skills. This instructional approach might thus be able to valuably contribute to the curricular requirement of providing medical students with the practical skills to perform and interpret clinical examinations. However, our findings suggest that it does not adequately address the students’ motivational needs i.e. going bedside to apply the skills acquired in a clinical context to feel well prepared for their future practice. During the ongoing pandemic medical students are faced with sparse possibilities for patient contact on the verge of becoming physicians. Therefore, medical teachers have to find creative ways to address their students’ needs while at the same time protecting their patients. Thus, in situations like the ongoing pandemic, when going bedside poses unusual risks, in-person practical “hands on” teaching sessions without patient contact can be valuable as an alternative strategy. Still, medical teachers should emphasize the clinical application of the practical skills acquired as much as possible e.g. by using case-based learning, patient videos or even real-time virtual patient contact.

Furthermore, in the ongoing pandemic, hybrid teaching formats might provide a pragmatic approach to the risks / benefits dilemma of bedside teaching. Integrating digital content developed during the pandemic into existing curricula could lead to didactic differentiation in the long term. Such future concepts should aim to enhance effectiveness of bedside teaching in view of limited possibilities and time windows for in-person teaching. In this context, flipped-classroom concepts, in which theoretical knowledge is a basis for in-person teaching with a focus on practical skills, might be powerful and should hence be the focus of future studies. For instance, e-learning formats could be used to prepare students e.g. regarding concepts and theoretical backgrounds of examination techniques before their bedside teaching. Moreover, such formats might also be valuable for students to recapitulate subject matter if necessary. This will not only aid efforts to best cope with the present pandemic situation, but also holds considerable potential to make bedside teaching more diverse and innovative.

## Data Availability

Data are available from the corresponding author (HH) upon request.

## Abbreviations

BF_10_: Bayes factor
CI: Confidence interval
e.g.: For example
i.e.: Id est
NRS: Numerical Rating Scale
SD: Standard deviation
T0: Baseline evaluation before the course
T1: Follow-up evaluation after the course
TUM: Technical University of Munich
